# An Optimized YOLOv11 Framework for the Efficient Multi-Category Defect Detection of Concrete Surface

**DOI:** 10.3390/s25051291

**Published:** 2025-02-20

**Authors:** Zhuang Tian, Fan Yang, Lei Yang, Yunjie Wu, Jiaying Chen, Peng Qian

**Affiliations:** 1 School of Transportation and Civil Engineering, Nantong University, Nantong 226019, China; 2333310011@stmail.ntu.edu.cn (Z.T.); 2118310009@stmail.ntu.edu.cn (L.Y.); 2233310004@stmail.ntu.cn (Y.W.); 2 College of Geographic Science, Nantong University, Nantong 226019, China; 2321110273@stmail.ntu.edu.cn

**Keywords:** YOLO, deformable convolution, dynamic convolution, concrete defect, digital optical images, spatial resolution

## Abstract

Thoroughly and accurately identifying various defects on concrete surfaces is crucial to ensure structural safety and prolong service life. However, in actual engineering inspections, the varying shapes and complexities of concrete structural defects challenge the insufficient robustness and generalization of mainstream models, often leading to misdetections and under-detections, which ultimately jeopardize structural safety. To overcome the disadvantages above, an efficient concrete defect detection model called YOLOv11-EMC (efficient multi-category concrete defect detection) is proposed. Firstly, ordinary convolution is substituted with a modified deformable convolution to efficiently extract irregular defect features, and the model’s robustness and generalization are significantly enhanced. Then, the C3k2module is integrated with a revised dynamic convolution module, which reduces unnecessary computations while enhancing flexibility and feature representation. Experiments show that, compared with Yolov11, Yolov11-EMC has improved precision, recall, mAP50, and F1 by 8.3%, 2.1%, 4.3%, and 3% respectively. Results of drone field tests show that Yolov11-EMC successfully lowers false and under-detections while simultaneously increasing detection accuracy, providing a superior methodology to tasks that require identifying tangible flaws in practical engineering applications.

## 1. Introduction

Given its exceptional mechanical qualities, durability, and affordability, concrete is one of the most widely used materials in construction engineering. It is extensively utilized in the building of diverse infrastructures [1]. Although concrete has strong compressive strength, it has inadequate tensile strength, which can be properly compensated for by mixing it with steel reinforcement, extending the structure’s service life [2]. However, exposed reinforcement, cracks, spalling, rust, whitening, delamination, and other defects may be present in concrete structures as a result of the combined impacts of several causes, including material quality, construction process, design flaws, environmental conditions, and incorrect usage. In addition to detracting from aesthetics, these flaws have the potential to create safety incidents such as building collapses and falls [3,4], which might cause large losses in terms of both persons and property. The construction unit can significantly lower the safety risk and extend the structure’s service life if it can identify defects early on and initiate corrective action [5].

The traditional manual inspection approach relies heavily on the inspectors’ understanding along with technical proficiency, demonstrating limits such as pronounced subjectivity and challenges in high-altitude inspections, which contribute to certain issues being undetected and unaddressed promptly [6]. The advancement of image processing and deep learning detection methods in recent years has been a great method to address the drawbacks of manual detection [7,8,9]. With the development of artificial intelligence, more and more researchers have started to use deep learning target detection algorithms to detect concrete defects. However, these researchers identify only one kind of defect in concrete structures or one scale of information, ignoring the diversity and complexity of defects in real projects, while the detection models they used in the past can lead to misdetection and under-detection in real engineering applications, which can seriously reduce the risk assessment level and affect the effectiveness of disaster prevention and mitigation [10,11].

We offer an effective technique in this research for locating flaws in concrete, which is based on YOLOv11 [12], to identify numerous defects on the surface of concrete structures. These are this study’s two contributions: (1) Our model replaces the traditional convolution with an enhanced deformable convolution, which can better handle complex shape features and greatly improved the capacity for generalization and the robustness of the model; and (2) by integrating C3K2 modules with a modified dynamic convolution approach, which adaptively adjusts convolution operations based on varying input characteristics, the model’s accuracy and adaptability in handling complex scenarios are significantly enhanced.

Here is how the paper is organized: Cutting-edge research on concrete defect detection is reviewed in Section 2. Our proposed model is described in Section 3, including the improved module as well as the advantages. The experimental part is described in Section 4, including the preparation of the experiment, the experimental environment, and the analysis of the results. The discussion is given in Section 5, and, finally, the conclusion is presented in Section 6.

## 2. Related Works

In the field of construction defect detection, deep learning has significantly advanced computer vision capabilities, enabling the efficient and accurate identification of structural issues. Among deep learning algorithms, the YOLO [13] (You Only Look Once) family has emerged as a popular choice for real-time detection tasks due to its simplicity and adaptability to resource-constrained environments. YOLO-based algorithms have been widely applied in various scenarios, including autonomous driving [14], security surveillance [15], and medical imaging [16], demonstrating their versatility and efficiency.

In the context of concrete structure maintenance, timely defect detection is critical for ensuring structural health. Traditional image processing techniques, such as edge detection [17] and feature extraction [18], have been surpassed by convolutional neural networks (CNN), which automate multi-level feature extraction and classification through end-to-end learning. CNN-based techniques, such as target classification [19], image segmentation [20], and target detection [21], have enhanced the accuracy and comprehensiveness of defect identification.

However, some of the existing studies have either focused on detecting a single defect type, such as cracks, or suffer from leakage and misdetection in the detection of multiple types of defects, which greatly limits their applicability in real-world engineering scenarios. BridgeNet combines techniques such as Swin Transformer, CARAFE upsampler, and migration learning to significantly improve damage recognition, but in experimental comparisons, BridgeNet’s derived models suffer from leakage and misdetection in detecting different types of defects [22]. SHM systems based on vibration-based structural system identification techniques can effectively monitor structural damage, but due to the limitations of the sensor installation work and the environment, these systems can lead to leakage during the monitoring of concrete defects [23].

The YOLO [13] family of target detection algorithms is popular for real-time detection tasks because it uses neural networks to predict both categories and bounding boxes simultaneously. Its simplicity, ease of implementation, support for multi-scale detection, and adaptability to new datasets make it particularly suitable for high-performance applications with resource constraints [24]. Recent advancements have further enhanced YOLO-based algorithms for specific tasks, such as concrete crack detection. For example, YOLOv8-CD improves feature extraction capability and detection accuracy while reducing computational complexity by combining visual attention networks (VAN), large separable kernel attention (LSKA), and the Ghost module [25]. The MOD-YOLO algorithm enhances detection accuracy and efficiency by incorporating deep separable convolutions, global sensory field pooling, and a coordinated attention mechanism, while minimizing computational costs [26]. Additionally, the R-YOLO v5 variant of YOLOv5 introduces angular regression variables and a novel attention mechanism to achieve high-performance detection with fewer computational resources, making it suitable for deployment on smaller devices [27]. Another lightweight deep learning approach significantly improves fine-grained feature extraction and gradient flow by reusing shallow features and optimizing feature convolution, providing an efficient real-time defect detection solution with reduced computational cost for mobile devices [28]. These improvements have significantly enhanced detection accuracy and inference speed, facilitating the integration of YOLO algorithms with UAVs for real-time, high-efficiency defect detection. In UAV-based concrete crack detection, methods such as background denoising and crack width calculation algorithms have been employed to improve detection efficiency for large scene images [29]. The DGAP-YOLO algorithm enhances detection accuracy and robustness by combining UAV-captured images with the DGC attention module and a low AFPN network [30], while the USSC-YOLO algorithm improves detection accuracy at different scales by incorporating ShuffleNet V2, Coordinate Attention Mechanism (CA), and Swin Transformer [31]. Despite these advancements, most studies focus on detecting a single defect type, such as cracks, and lack multi-scale comparative analyses, which may limit the broader application of these models in real-world engineering, where multi-defect detection across diverse environments is required.

In practical detection processes, the diverse shapes of concrete defects and the complexity of their surrounding environments are vital factors influencing the detection efficiency. In Section 3, these two aspects are mainly taken into consideration.

## 3. Proposed Method

This study proposes an effective YOLOv11-based concrete defect target detection algorithm: YOLOv11-EMC(Efficient Inspection Method for Multiple Defects on Concrete Surfaces), which is created to address the fundamental issue of identifying surface flaws in concrete in intricate settings, especially for the application scenarios where the defects are complex and diverse in morphology, unevenly distributed, and with strong background interference, and to provide an efficient and accurate detection solution. First, we introduce dynamic convolution incorporating the ECA attention mechanism as a way to improve the robustness and generalization of the model to defects of different shapes. Then, the enhanced dynamic convolution with an embedded SE attention mechanism significantly reduces the computational cost of the model, realizing efficient detection with low computational cost.

### 3.1. YOLOv11

**Figure 1:**
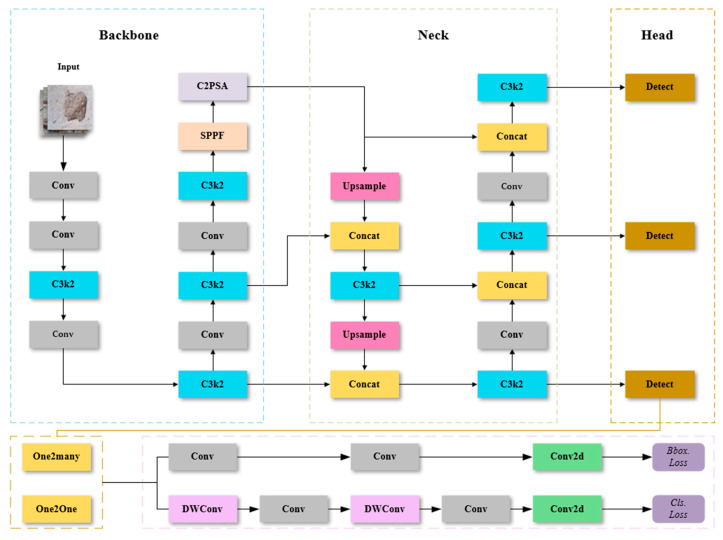
YOLOv11 network structure diagram.

YOLOv11, like YOLOv8 [32] and YOLOv5 [33], was also proposed by the Ultralytics team. Compared to their previous work, YOLOv8, the main improvement points of YOLOv11 are the introduction of the C3k2 mechanism, the addition of two DWConvs to the classification detector header in the original decoupling header, and the addition of an SPPF followed by a layer of C2PSA. YOLOv11 uses the same detection head structure as YOLOv10 [34]. The network architecture of YOLOv11 is shown in Figure 1.

### 3.2. YOLOv11-EMC Network Infrastructure

**Figure 2:**
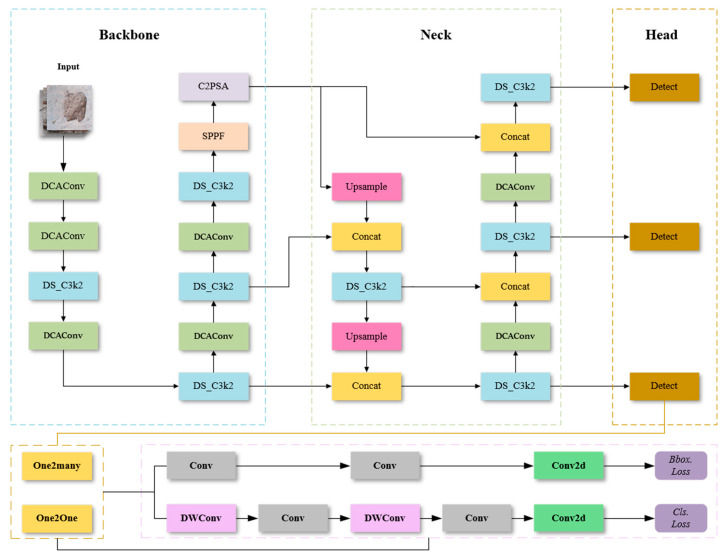
YOLOv11-EMC network structure diagram.

YOLOv11-EMC uses an enhanced deformable convolution module, which combines deformable convolution with ECA attention: the Deformable Convolution with ECA Attention module, from now on referred to as the DCA module. The DCA module improves the flexibility and efficiency of YOLO’s traditional network structure when dealing with complex features, especially when dealing with changing object shapes and complex background information in an image. It can extract the key features in the image more accurately and improve the performance of the model. In addition, our model also incorporates the C3K2 module into an enhanced dynamic convolution module, i.e., the dynamic convolution and SE attention are fused into a new module: the Dynamic Convolution with SE Attention module, hereafter referred to as the DS_C3K2 module, which improves the neural network’s spatial and channel feature extraction while reducing computational complexity. The method performs particularly well in tasks requiring high-accuracy performance, especially in concrete defect detection. The network architecture of YOLOv11-EMC is shown in Figure 2.

### 3.3. DCA Module

#### 3.3.1. Deformable Convolution

In a traditional convolution operation, convolution kernels sample the input feature map according to a fixed grid structure. Each convolution kernel position corresponds to a fixed set of sampling points, and the positions of the sampling points are fixed relative to the center point. For targets with irregular shapes or non-rigid structures, this fixed sampling approach cannot effectively capture features. The standard convolution operation can be written as follows for any point *p*_0_ on the input feature map:

(1) $$y(p_{0}){=}_{\;p_{n}\;\in \;R}\sum w(p_{n})\cdot x(p_{0}+p_{n})$$

*P*_0_ is the current center position of the convolution kernel; *p_n_* is the offset of each point in the convolution kernel with respect to the center point; *w*(*p_n_*) is the weight of the corresponding position of the convolution kernel; *x*(*p*_0_ + *p_n_*) is the element value corresponding to the (*p*_0_ + *p_n_*) position on the input feature map; *y*(*p*_0_) is the element value corresponding to position *p*_0_ on the output feature map.

**Figure 3:**
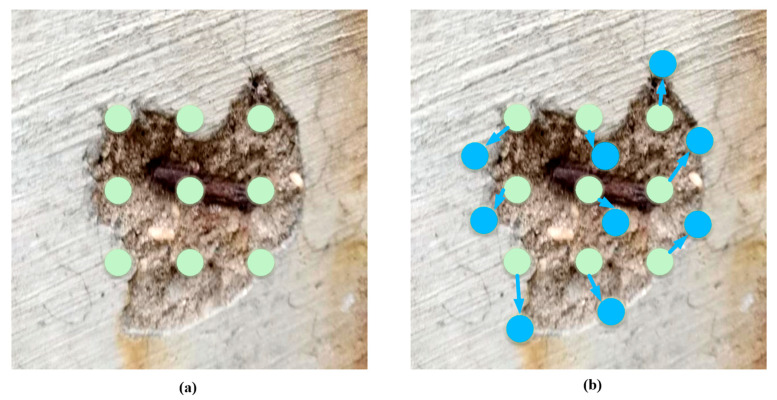
Regular convolutional kernel vs. deformable convolutional kernel plots: (**a**) regular convolutional kernel; (**b**) deformable convolutional kernel. The green circle represents the arrangement of regular convolution, the blue circle represents the arrangement of deformable convolution learned with offset, and the arrow represents the direction of offset.

The key to deformable convolution [35] is to introduce learnable offsets for each convolutional sample point. An extra convolutional network is used to learn these offsets, and it is possible to continuously modify it while training. In this way, the sampling locations of the convolutional kernel are no longer fixed but are dynamically determined by these offsets, allowing for greater flexibility in capturing irregular shapes and structural features. The visualization principle is shown in Figure 3; the principle of deformable convolution is based on a network learning offset that causes the sampling points of the convolution kernel at the input feature map to be shifted, focusing on the region or target we are interested in.

The deformable convolution operation can be represented by the following equation:

(2) $$y(p_{0}){=}_{\;p_{n}\;\in \;R}\sum w(p_{n})\cdot x(p_{0}+p_{n}+∆p_{n})$$

∆*P_n_* is the offset learned through the offset learning module. Compared with the ordinary convolution operation, this convolution operation with the added offset is more capable of eliminating the interference of background noise and obtaining more useful information.

The computational steps of deformable convolution are as follows:

(1) Offset learning: to produce an offset ∆*p*, a standard convolutional layer is first applied to the input feature map;
(2) Dynamic sampling: for each convolutional position, the sampling points are adjusted using the offset ∆*p* corresponding to the position. Since the offset sampling points are generally not integers, the eigenvalues of these non-integer positions must be obtained via bilinear interpolation;
(3) Convolutional computation: to output the offset ∆*p*, a regular convolutional layer is applied to the input feature map after the convolutional layer has been applied.

Since ∆*p* is usually the data in floating point form obtained after learning, *x*(*p*_0_ + *p_n_* + ∆*p_n_*) needs to be determined by bilinear interpolation as follows:

(3) $$x(p)=\sum_{q}G(q,p)\cdot x(q)$$

(4) G(q,p)=g(qx,px)⋅g(qy,py)

where *g*(*a*, *b*) = *max*(0, 1 − |*a* − *b*|); G(*q*, *p*) is non-zero only for a few *q_s_*.

In the formula, *q* is the nearest integer pixel position in the p-domain and *G* is a bilinear interpolation kernel consisting of two one-dimensional kernels. The aforementioned formula demonstrates that the weighted sum of the adjacent pixel points determines the pixel value at the interpolation point location, and the weight of each pixel point is determined according to the distance from the horizontal and vertical coordinates of the interpolation point. Max (0, 1) is the constraint that the interpolated point’s distance from the nearby pixel points must never exceed 1 [35].

**Figure 4:**
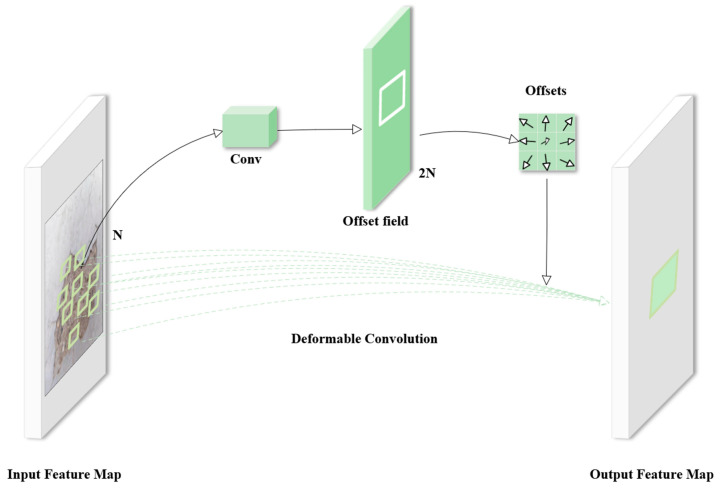
Schematic of the 3 × 3 deformable convolution module.

As shown in Figure 4, the offsets are generated using additional convolutional learning, which is not the same as the final convolutional operation to be performed. For the input feature map, the region size of the convolution kernel can be 3 × 3, at which point N = 9. After the offset learning process, the convolution kernel region of the feature map becomes 2N (offset field in Figure 4) because the con-volution kernel learns the offsets in the x-direction and y-direction, respectively.

Deformable convolution enables the convolution kernel sampling points to adaptively deform according to the morphological features of defects by introducing learnable spatial offset parameters. For example, in crack detection, the sampling points can be arranged in an extended arrangement along the main direction of the crack to enhance long-range feature extraction; facing the irregular boundary of the spalled region, the offset can drive the convolution kernel to focus on the region of mutant gradient. This dynamic deformation mechanism not only breaks through the rigid structural limitation of traditional convolution but also effectively suppresses the interference of concrete surface texture noise through the adaptive adjustment of feature space.

#### 3.3.2. Enhanced Deformable Convolution

**Figure 5:**
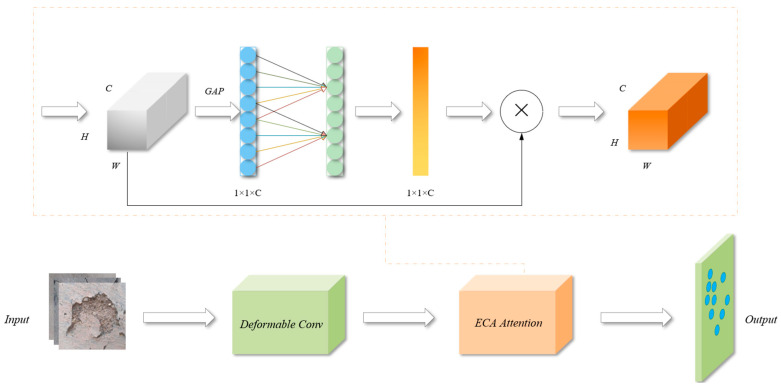
DCA module schematic.

In order to improve the adaptability of convolutional neural networks to complex geometric transformations as well as to focus on important features, an enhanced version of deformable convolution is proposed, called the DCA module. The structure of the DCA module is shown in Figure 5. The core idea of the DCA module is to take the feature maps generated by deformable convolution and generate the attention weights through the ECA module to enhance the important features and suppress the unimportant ones, which in turn improves the model performance.

The main idea behind ECA is to obtain inter-channel dependency by using a convolution operation rather than a fully connected layer [36]. This reduces the number of parameters and the computational effort. It uses a 1D convolution to accomplish more effective channel weight computation. The working schematic is as follows:

The fundamental procedure is as follows:

(1) Global average pooling: to obtain a feature vector of channel dimension, global average pooling is first carried out for each channel of the input feature map. The function of this step is to perform global statistics on each channel to obtain a global description of the channel.
(2) 1D convolution operation: next, ECA captures the dependencies between channels by a 1D convolution (instead of a fully connected layer). The kernel size of this 1D convolution is adaptively determined based on the number of input channels, which reduces the computational effort while obtaining effective channel attention.
  (5) $$k=\psi (C)=\left|\right.\frac{\log2(C)}{\gamma }+b\left.\right|$$
  where *C* is the number of channels and γ and b are the hyperparameters used to regulate the size of the convolution kernel.
(3) Sigmoid activation: the output of the 1D convolution is subjected to sigmoid activation to obtain the weight coefficients for each channel. This limits the weight of each channel to between 0 and 1, indicating the importance of that channel.
(4) Channel weighting: finally, the obtained weight coefficients are multiplied channel-by-channel with the original input feature map to complete the weighting operation of the channel attention.

The way the DCA model operates is as follows:

(1) The input feature map x first enters the Deformable Convolution module. In the Deformable Convolution module, the offset is first calculated through the offset_conv layer, which is used to adjust the sampling position of the regular convolution for deformation convolution.
(2) The Deformable Convolution feature map enters the ECA module. In the ECA module, the input feature map is first compressed into a one-dimensional vector by global average pooling and then reshaped to (b, c), where b is the batch size and c is the number of channels.
(3) Next, a one-dimensional convolution is applied after the dimensionality expansion, and then the attention weight is obtained by the sigmoid activation function.
(4) Finally, the attention weights are reshaped and multiplied element-by-element with the input feature maps, and the attention weights are applied to the Deformable Convolution feature maps to highlight important features. The feature map processed by the ECA attention module is returned as the final output of the DCA Conv module.

The DCA model is able to extract key features in the image more accurately and improve the performance of the model with the following advantages: (1) Enhanced robustness: On the one hand, deformable convolution techniques introduce flexibility by dynamically adjusting the sensory field of the convolution kernel, allowing it to better handle geometric deformations and local changes in the input image. On the other hand, ECA adaptively allocates weights to each channel according to its significance, improving the model’s capacity to concentrate on important features. When these techniques are combined, they synergistically enhance the robustness of the model. (2) Improved Accuracy: The integration of ECA with deformable convolution significantly improves the accuracy of the model and enhances its generalization capabilities across a wide range of engineering tasks.

### 3.4. DS Module

#### 3.4.1. Dynamic Convolution

**Figure 6:**
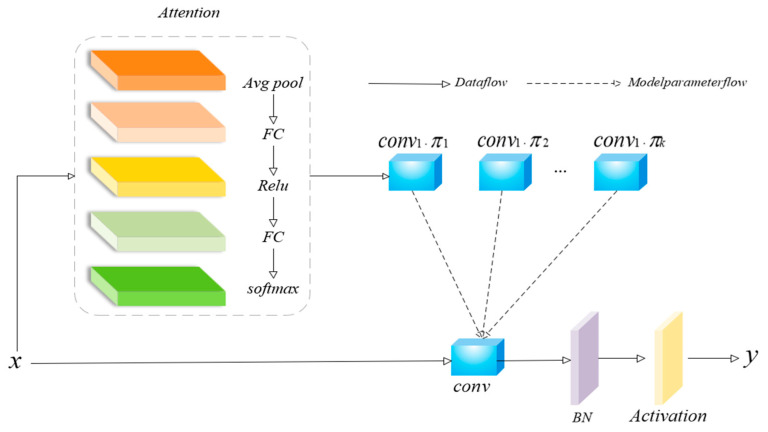
Dynamic convolution schematic.

The fundamental concept of dynamic convolution [37] is the use of multiple convolution kernel sets in a convolution operation, with the kernels being adaptively weighted and summed based on the input feature content. The working diagram of dynamic convolution is as follows:

Figure 6 illustrates the structure and working principle of dynamic convolution. The structure realizes dynamic feature extraction by introducing an attention mechanism to adaptively generate a set of convolution kernel weights for each input feature. It is mainly divided into the following parts:

- Attention Module
  (1) The input feature map first enters the Attention Module, and a compact feature vector is extracted by global average pooling (Avg Pool).
  (2) The ReLU activation function is added after the first fully connected layer to enhance the model’s capacity for nonlinear expression after the feature vector sequentially moves through two fully connected layers (FC).
  (3) The last step is to use a softmax activation function to create a vector of weight coefficients. The control over the selection of the various convolutional kernels is normalized by softmax, which makes sure that the output weight values fall between 0 and 1 and that the sum of the weights is 1.
- Dynamic Convolutional Kernel Selection
  (1) Figure 6 shows multiple parallel convolutional kernels (e.g., conv1, conv2,... convn), which represent different feature extraction modes and are able to learn different features separately.
  (2) A vector of weight coefficients weights the output of each convolutional kernel, and the output feature map of each convolutional kernel is adjusted according to the weights, and finally the output features of the dynamic convolution are generated by weighted summation.
  (3) This weighted summation mechanism ensures that the network can automatically adjust the combination of convolution kernels according to the differences in the input features and has the ability to perform dynamic feature extraction.
- Final Convolution Operation
  (1) The weighted output feature map is then passed through a standard convolutional layer (Conv) to further extract features, and then through batch normalization (BN) and activation functions to generate the final output.
  (2) The activation function provides the model with stronger nonlinear capabilities to improve the characterization of complex features, while batch normalization speeds up convergence and increases training stability.
- Data Flow and Parameter Flow
  (1) The solid arrows indicate the forward transfer path of the input data, while the dashed arrows indicate the action path of the model parameters.
  (2) The weights generated by the Attention Module are used to control the participation of different convolutional kernels, thus indirectly affecting the generation of the final feature map.

In traditional convolution, the whole input feature map x is subjected to a constant receptive field R, and the convolution kernel parameter *w*(*q*) is fixed:

(6) $$y(p)=\sum_{q\in R}x(q)\cdot w(q)$$

where *y*(*p*) is the output, *x*(*q*) is the input feature, *w*(*q*) is a fixed convolution kernel, and *R* is the receptive field of the convolution kernel. In conventional convolution operations, the parameters of each convolution kernel are fixed. For a given input feature map, the convolution kernel is shifted over the entire input and applied to a fixed local region to extract features.

Dynamic convolution, on the other hand, dynamically generates weights *w*(*q*) from the feature function *f*(*x*), and the choice of convolution kernel varies with the input features:

(7) $$y(p)=\sum_{q\in R}x(q)\cdot w(q,f(x))$$

By adaptively modifying the feature extraction process, dynamic convolution greatly enhances the network’s expressiveness and adaptability for a variety of input scenarios.

In concrete defect detection, complex environments may contain different weather variations, different texture variations, and a diversity of defect morphologies, which often lead to poor extraction results when processed by traditional neural networks. Dynamic convolution can dynamically adjust the parameters of the convolution kernel according to the specific scene inputs, thus improving the recognition of various defects. For defects such as gaps, holes, or spalling on the concrete surface, dynamic convolution is able to maintain focused detection accuracy under incomplete signals, different defect morphologies, and environmental noise, which enhances the stability and robustness of the model.

#### 3.4.2. Enhanced Dynamic Convolution

To enable the dynamic convolution module to better adapt to different data distributions and feature patterns, we incorporate the SE attention mechanism, called DS module. By adding the SE attention mechanism, the DS module learns the attention weights between channels, improves the robustness and generalization ability of the model, increases the performance ability of the model in various scenarios, and suppresses the unimportant channel features while enhancing the important channel features to better adapt to various data distributions and feature patterns.

The SE module (Squeeze-and-Excitation Module) is a channel attention mechanism that improves the feature selection capabilities of convolutional neural networks by adaptively allocating weights to each channel in order to suppress irrelevant features and enhance important features. Squeeze and Excitation are primary components of the SE module [35]. The principle of operation is as follows:

(1) Squeeze

To compress the input feature map from *H* × *W* × *C* to 1 × 1 × *C*, global average pooling is first applied to each channel. Here, *H* and *W* stand for the feature map’s height and width, and *C* for the number of channels. As a result, a value representing the global semantic information for each channel will be output. This step aims to create a description of each channel’s global features.

(8) $$Zc=\frac{1}{H\times W}\sum_{i=1}^{H}\sum_{j=1}^{W}xc(i,j)$$

where *x_c_*(*i*, *j*) denotes the value of the input feature map at the (*i*, *j*) position on channel *c*.

(2) Excitation

In order to minimize computational effort, the first fully connected layer typically cuts the channel dimensions in half. The second fully connected layer creates weights using sigmoid activation and returns to the initial channel count. Each channel’s importance is indicated by these weights, and the more significant a channel is, the higher its value.

(9) $$s=\sigma (W_{2}\cdot \delta (W_{1}\cdot z))$$

where *W*_1_ and *W*_2_ are the weight matrices of the fully connected layer, the ReLU activation function, and the sigmoid activation function.

(3) Scale

Ultimately, the acquired weights are multiplied by the original feature map channel by channel to accomplish the adaptive rescaling of the features. In other words, the output of the corresponding feature map channel increases with increasingly higher channel weights, and vice versa.

(10) $${\tilde{x}}_{c}=x_{c}\cdot s_{c}$$

where *S_c_* is the weight of channel *c*; and *x_c_* represents the data of channel *c* in the input feature map.

The DS module operates as follows:

(1) Input and initialization:

The DS module receives the input tensor, which has the shape. The DS module first calls the initialization function of the parent class C3k2_DynamicConv for some basic initialization of the convolutional layers and modules.

(2) Parent module processing (C3k2_DynamicConv):

Then, the output y is sliced into two parts along the channel dimension and then sequentially processed by a series of Bottleneck modules, and, finally, all the results are stitched together in the channel dimension, and then the output is obtained by the convolutional layer.

(3) SE attention mechanism processing:

In the forward function of the DS module, the forward function of the parent class is called first to process the input and obtain the intermediate results. Then, the intermediate result is input to the SE attention module. The SE attention module compresses the feature map into a vector through global average pooling, then learns the attention weights between channels through two fully connected layers, and finally multiplies the attention weights with the original feature map to enhance the features of the important channels and suppress the features of the unimportant channels.

(4) Output:

The feature maps processed by the SE attention module are used as the final output of the DS module.

**Figure 7:**
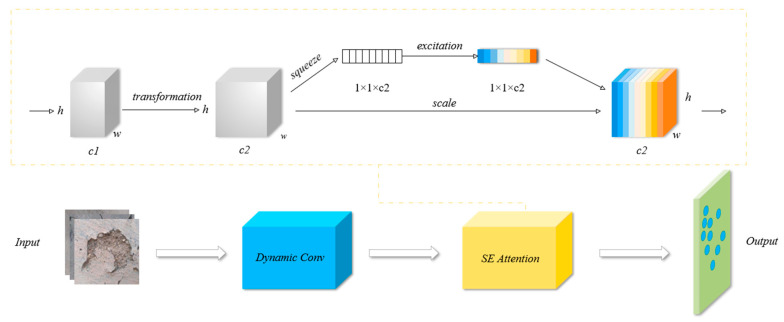
DS module schematic.

The structure of the DS module is shown in Figure 7. The advantages of the DS module are as follows: (1) Adaptive feature extraction: DynamicConv generates convolution kernels dynamically and flexibly modifies the kernels’ weights and shapes in accordance with the various input feature maps in order to capture a variety of features. However, by adaptively relabeling channel features, the SE module allows the network to more effectively concentrate on key feature channels. This combination enhances the model’s capacity to depict intricate features. (2) Better model performance: The combination can improve the model’s learning effect and accuracy on particular tasks, particularly when working with complex and diverse input data. (3) Optimization calculations: DynamicConv can produce convolutional kernels dynamically based on input, which minimizes the need for fixed convolutional kernels. It can enhance the feature representation capability without appreciably adding more parameters and computation amounts when used in conjunction with the SE module’s channel recalibration.

## 4. Experiment

### 4.1. Experimental Environment

The information of the experimental environment parameters of this study is shown in Table 1.

The main hyperparameters for the training set are as shown in Table 2.

We select the more common hyperparameter candidates and choose the optimal combination based on the experimental results to ensure efficient and accurate results.

### 4.2. Dataset

**Figure 8:**
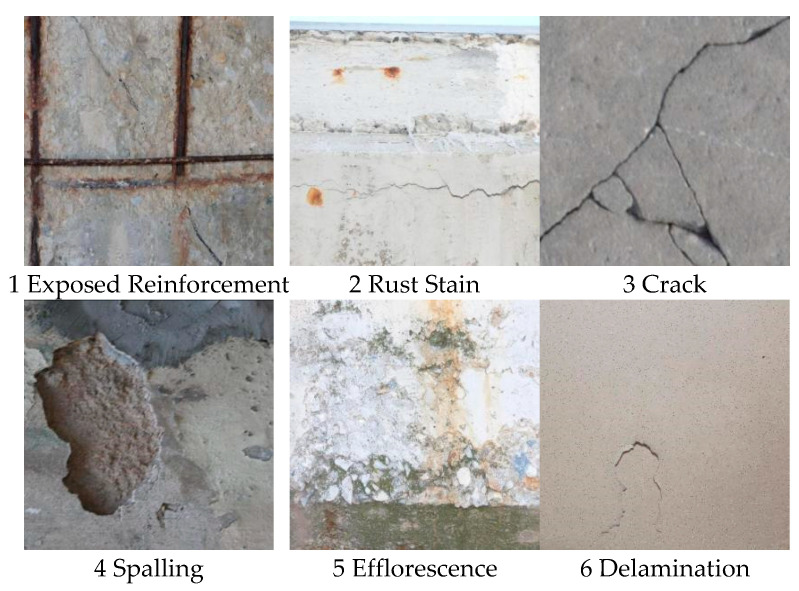
Examples of six different defects from the dataset.

This paper uses data from publicly available online datasets, including 7363 photos of concrete flaws found during construction [38]. These flaws can be classified into six categories: whitening, cracks, exposed reinforcement, rust, or delamination. Using an 8:1:1 ratio, these images are separated into training, validation, and test sets. Examples of the six deficiencies in the dataset are shown in Figure 8.

### 4.3. Evaluation Metrics

This paper uses True Positive (TP), True Negative (TN), False Positive (FP), and False Negative (FN) as the main indicators of object detection. The YOLOv11-EMC network and other networks’ accuracy in the crack detection task can be evaluated using these metrics. Accurate predictions of positive and negative cases are indicated by TP and TN, respectively, while inaccurate predictions of positive and negative cases are indicated by FP and FN.

(11) $$precision=\frac{TP}{TP+FP}$$

The precision is the proportion of true positive samples among the detected targets.

(12) $$recall=\frac{TP}{TP+FN}$$

The recall is the proportion of real targets that are detected.

(13) $$AP=\int_{0}^{1}p(r)dr$$

Current standards define the AP calculation as the region bounded by the x-axis and the interpolated PR curves. The AP (average precision) for each category is computed and averaged under various IoU [39] (intersection and concurrency ratio) thresholds to produce mAP. The trained model’s ability to detect all categories is measured by mAP, whereas the AP value is only calculated for one category. Assuming K categories, the map’s calculation formula is as follows:

(14) $$mAP=\frac{\sum_{i=1}^{K}{AP}_{i}}{K}$$

*F*_1_ is a composite metric used to weigh the precision and the recall, reflecting the model’s balance of handling different detection accuracies and recall rates.

(15) $$F_{1}=2\times \frac{precision\times recall}{precision+recall}$$

### 4.4. Results and Analysis

#### 4.4.1. Comprehensive Performance Indicators

We used Yolov11 as the baseline model and tested the hyperparameter optimization for the candidate values given in Table 2, using accuracy and test duration as evaluation metrics, and selected the optimal hyperparameter combination based on the test results, as shown in Table 3.

The thorough evaluation for the concrete defect detection task includes not only the widely used mAP (mean average precision), which calculates the average of the model’s precision at various IoU (intersection over union) thresholds, reflecting the model’s accuracy over a wide range of defect types, but also the precision and recall, the former of which assesses the proportion of samples with correct positive predictions and the latter of which assesses the proportion of positive samples found by the model. The computational efficiency and complexity of the model are then assessed using the number of parameters and GFLOPs (number of floating point operations per second), which aids in comprehending the model’s viability and hardware requirements for deployment. Finding a fair balance between accuracy, speed, computation, and complexity to satisfy the real application requirements is another benefit of carefully examining these metrics. It also aids in optimizing the model’s performance overall.

Table 4 shows the training parameters and results of different target detection algorithms. According to the experimental results, the comprehensive performance of YOLOv11-EMC is better than other models. First, the detection accuracy of YOLOv11-EMC is much higher than that of the YOLO series models, with a precision (P) of 0.81. The recall (R) is 0.596 and 0.666 in the mAP50-95 (mean accuracy) metric, which is second only to ResNet50-Faster R-CNN. Second, the computational consumption of YOLOv11-EMC is also relatively smaller, and, despite the fact that YOLOv11-EMC has more parameters than the same series models, the 3217730, it still maintains the same GFLOPS as YOLOv11n—6.3, which achieves a balance between accuracy and efficiency, creating the possibility of real-time detection in real engineering.

It is worth noting that the computational consumptions of ResNet50-Faster R-CNN and VGG16-SSD are much larger than those of the models of the YOLO series, which ensures a certain degree of detection accuracy, but our study is mainly aimed at real-time detection in real engineering, and the extremely large computational consumptions of ResNet50-Faster R-CNN and VGG16-SSD are obviously not sufficient for this requirement, despite their ideal accuracy. Therefore, we will focus on the YOLO family of models in the following sections.

#### 4.4.2. Robustness and Adaptability

Robustness is a crucial component in the training of concrete defect detection models, as it guarantees that the model can continue to operate effectively and steadily even when dealing with noise, variations in image quality, and various defect types. Even in the face of shifting data distribution and diverse environments, a robust model can enhance its capacity for generalization, prevent overfitting, and precisely identify a range of concrete flaws. Furthermore, robustness enhances the model’s capacity to adjust to faulty data in practical applications. Adopting data enhancement, regularization techniques, and noise injection during the training phase can effectively increase the model’s robustness. This ensures that the model has higher accuracy and reliability in the actual detection task and improves its adaptability against complex scenes and changes in the environment. Consequently, robustness is the primary characteristic that guarantees the concrete defect detection model’s high stability and adaptability in the complex application environment, in addition to being the key to optimizing its performance.

The mean average precision (mAP) values of the various models for six different types of concrete defects—exposed reinforcement, rust, cracks, spalling, whitening, and delamination—are compared in the above table. The YOLOv11-EMC model exhibits good detection performance for every type of defect.

In particular (as shown in Table 5), YOLOv11-EMC ties with YOLOv8n as the best with an mAP of 0.911 for exposed reinforcement detection. With an mAP of 0.467 for rust detection, YOLOv11-EMC outperformed the other models by a significant margin. Furthermore, YOLOv11-EMC had the highest mAPs of any model in crack and spalling detection, with mAPs of 0.607 and 0.787, respectively. YOLOv11-EMC leads the others in whitening detection with an mAP value of 0.354, even though the mAP of all the models is relatively low. YOLOv11-EMC’s mAP value, which is much higher than that of the other models, reaches 0.872, particularly in the stratification detection task.

#### 4.4.3. Detection Accuracy Indicators

Precision (P), recall (R), and mean average precision (mAP) are the three primary metrics used to evaluate model performance in target detection tasks. By concentrating on various facets of detection quality, these metrics offer a thorough evaluation of the model’s capacity to identify and categorize targets. Precision highlights the significance of reducing false positives in circumstances where misclassification can have major repercussions by quantifying the percentage of accurate predictions across all prediction instances. Recall, on the other hand, highlights the model’s capacity to minimize missed detections by calculating the percentage of actual objects that are correctly identified. A comprehensive evaluation of the model’s detection accuracy is provided by the mean accuracy (mAP), which combines the trade-off between precision and recall at various confidence thresholds.

**Figure 9:**
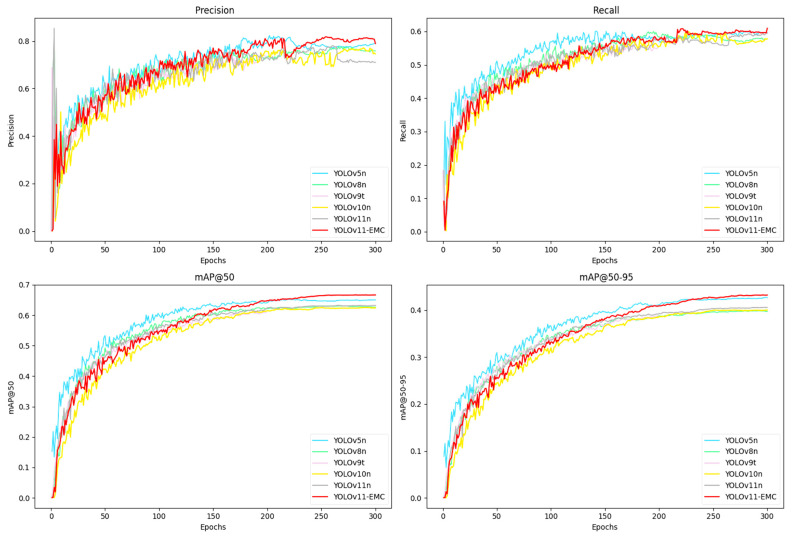
Comparison of the detection accuracies of different models during the training process.

Figure 9 illustrates the accuracy performance of the different models, which shows the key performance metrics of various YOLO models during training, including precision, recall, mean accuracy at IoU = 0.5 (mAP50), and mean accuracy at IoU ranges of 0.5–0.95 (mAP 50–95). Our model outperforms the other models.

Above all, the precision and recall curves of the YOLOv11-EMC model show a continuous upward trend and consistently high values throughout the late training phase, which suggests that the model has a strong ability to discriminate between positive and negative samples for identification. This suggests that the model can provide theoretical and empirical support for further research and practical applications and has considerable potential and adaptability in specific defect detection tasks.

#### 4.4.4. Field Testing

In our field tests, we used a DJI Air 3 UAV to capture a range of defective concrete surface images, including both near and far views, to simulate real-world conditions. The inference results show that our proposed model, YOLOv11-EMC, outperforms other models in terms of detection accuracy and robustness across different image scales. This demonstrates that YOLOv11-EMC is effective in detection, regardless of the scale or distance of the objects in the images, highlighting its reliability for practical applications.

**Figure 10:**
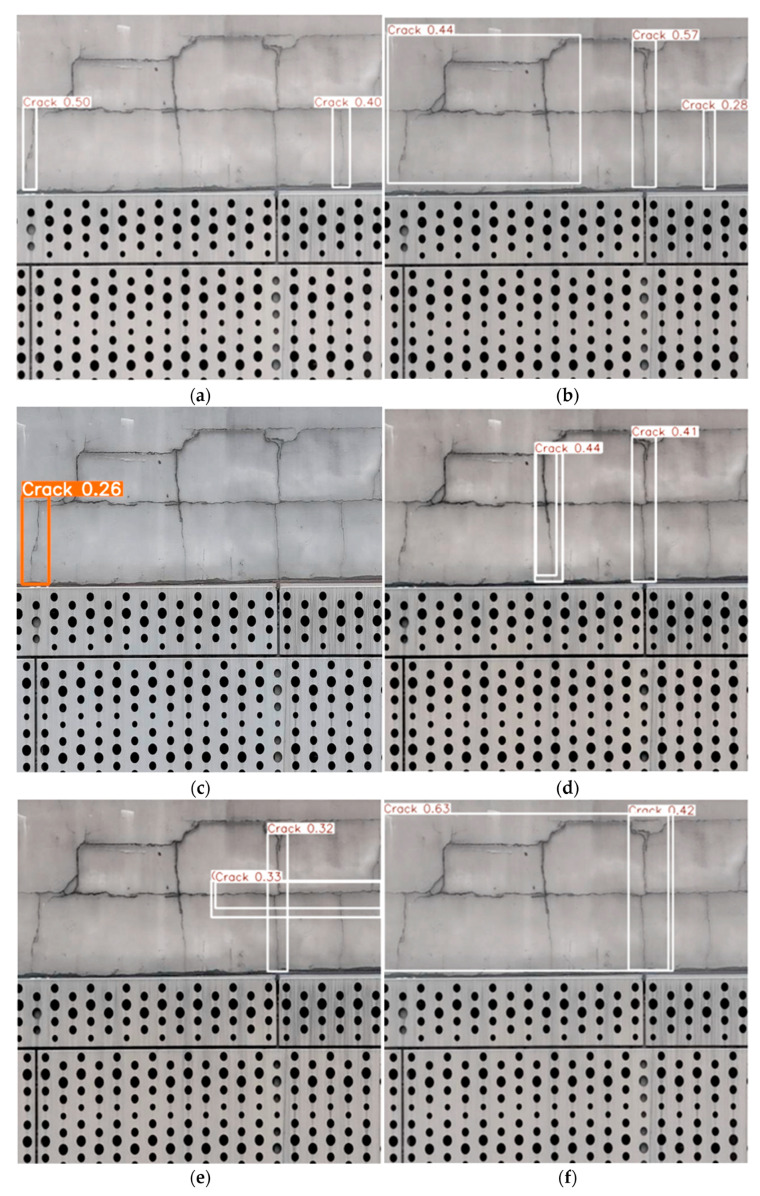
Comparison of the inference performance of different models in near views: (**a**) YOLOv5n; (**b**) YOLOv8n; (**c**) YOLOv9t; (**d**) YOLOv10n; (**e**) YOLOv11n; (**f**) YOLOv11-EMC.

As shown in Figure 10, in the near-view image inference, YOLOv11-EMC demonstrates the smallest leakage area and the highest inference accuracy of 63%. This indicates that the model excels in accurately locating and recognizing defects in close-range scenes, with reduced false negatives and enhanced precision in detecting fine details.

**Figure 11:**
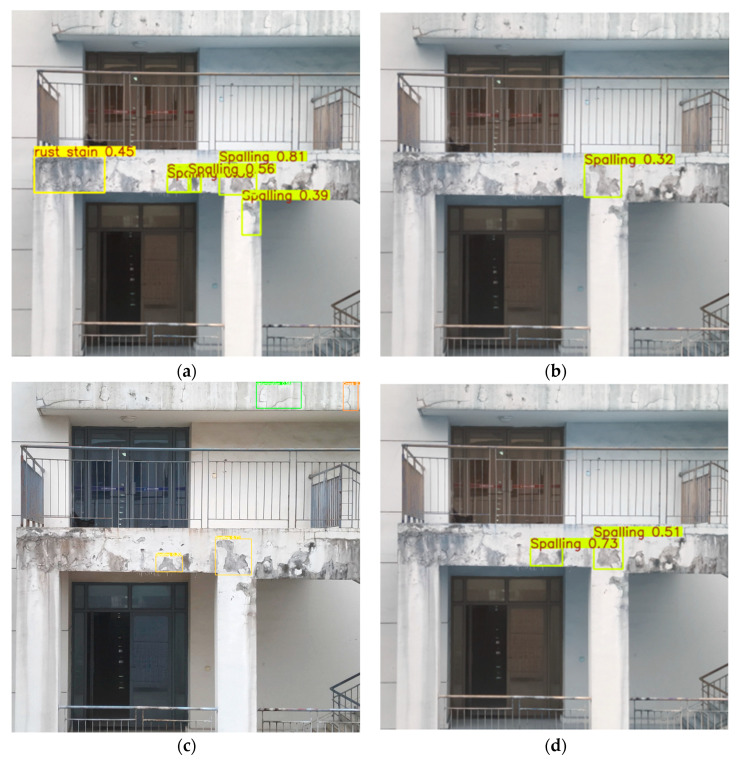

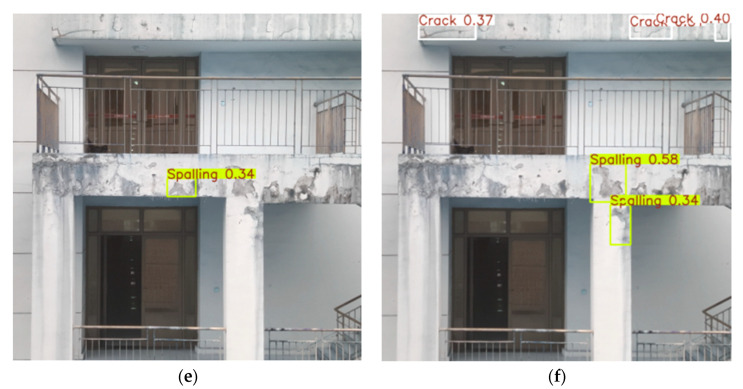
Comparison of the inference performance of different models in far views: (**a**) YOLOv5n; (**b**) YOLOv8n; (**c**) YOLOv9t; (**d**) YOLOv10n; (**e**) YOLOv11n; (**f**) YOLOv11-EMC.

As shown in Figure 11, YOLOv11-EMC detected the second-highest number of defective parts in the far-view image inference, identifying five defective regions, compared to YOLOv5. While YOLOv5 successfully detected some defects, it struggled with overlapping detection frames and misclassification, such as mistakenly identifying benign surface stains as rust defects. These issues led to a decrease in overall accuracy and reliability. In contrast, YOLOv11-EMC not only minimized such errors but also demonstrated superior performance in defect classification. The model consistently maintained high accuracy, distinguishing between different defect types with greater precision, thereby providing clearer and more reliable inspection results.

The exceptional robustness and generalization capabilities of YOLOv11-EMC are evidenced by its superior performance in both near-field and far-field image inference. This consistent detection ability across varying spatial resolutions and perspectives highlights the model’s adaptability and utility in real-world applications.

#### 4.4.5. Classification Verification

To comprehensively evaluate the model’s effectiveness, we conducted experiments using images from six distinct categories of concrete defects, comparing YOLOv11-EMC with the original YOLOv11 framework. The dataset incorporated diverse real-world conditions, including variations in lighting, texture, and environmental complexity.

**Figure 12:**
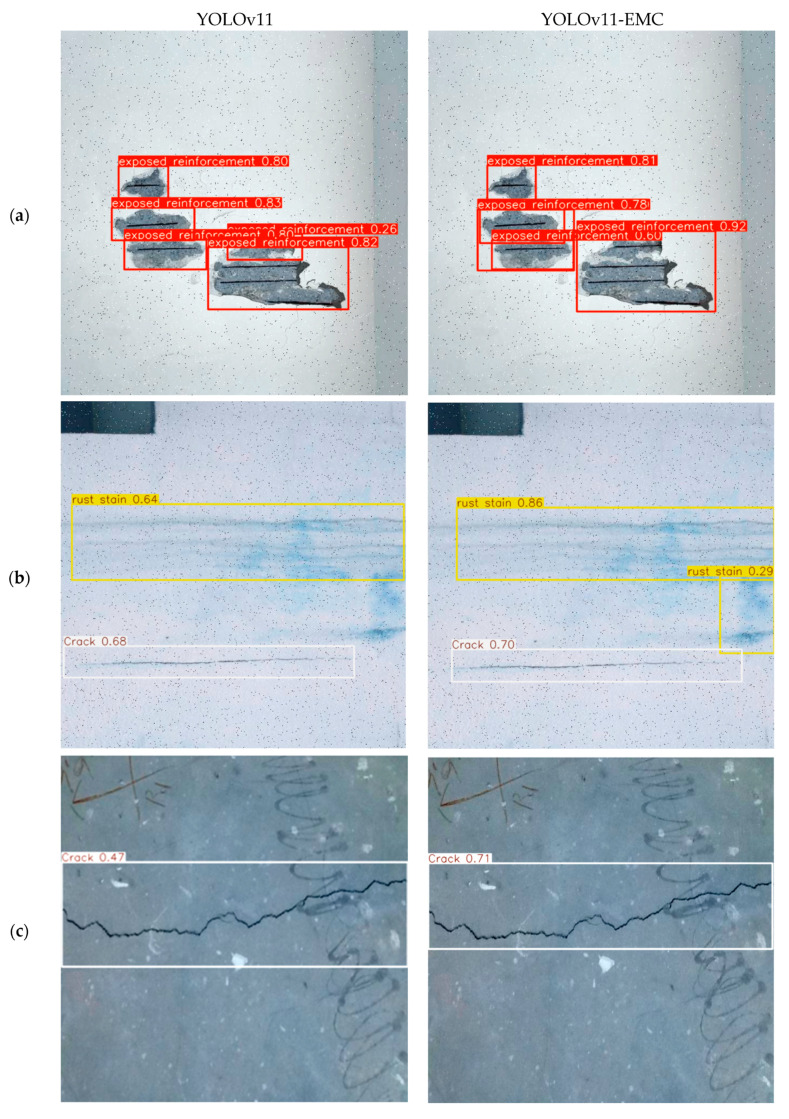

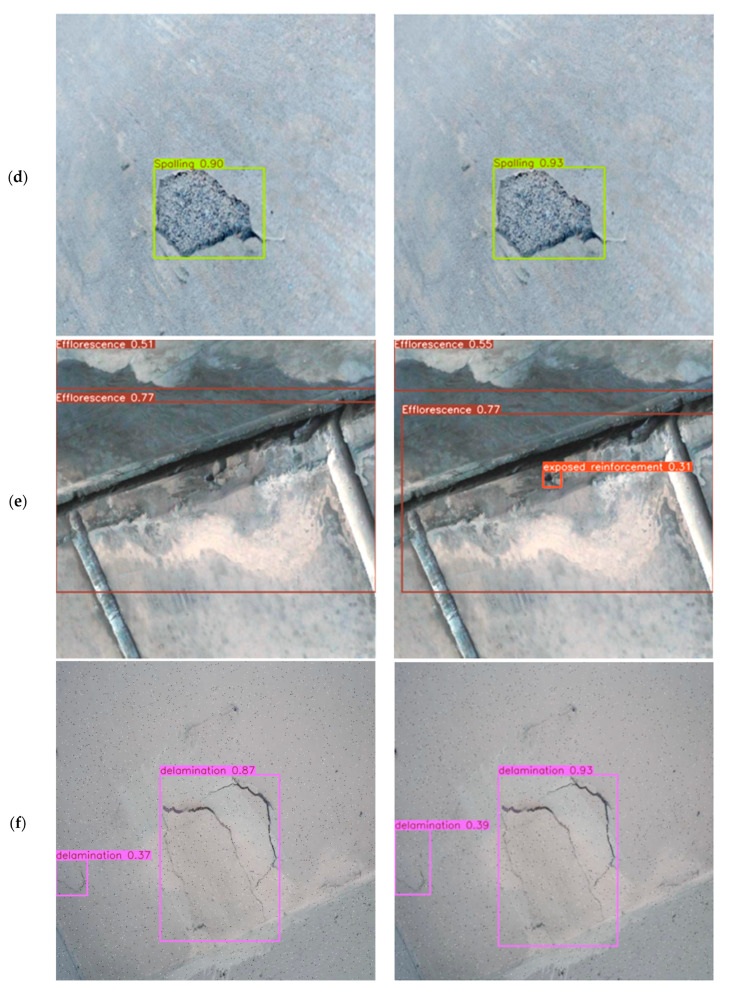
Comparison of the performance of different defect inferences: (**a**) exposed reinforcement; (**b**) rust stain; (**c**) crack; (**d**) spalling; (**e**) efflorescence; (**f**) delamination.

Figure 12 shows the results of YOLOv11 and YOLOv11-EMC for different defective pictures tested. Based on the comparative analysis of the six defect detection result graphs, YOLOv11-EMC has different degrees of improvement compared to the original YOLOv11, with the highest degree of improvement in crack detection, which reaches 24%, and the lowest degree of improvement in whitening, which is only 4%. In a comprehensive view, YOLOv11-EMC exhibits excellent detection accuracy, strong multi-category recognition capability, and a low false detection rate.

Table 6 illustrates how our model performs better than YOLOv11n in terms of accuracy throughout the inference validation procedure, where precision (p) improves by 8.3%, recall (r) improves by 2.1%, mAP improves by 4.3%, and the F1 score increases by 3%, with no increase in computational resources. This provides a high-quality solution for the detection of devices that have limitations on computational resources but require a high level of accuracy. Notably, YOLOv11n’s frames per second (FPS) are higher than ours, indicating that our model’s real-time performance still requires work.

## 5. Discussion

The YOLOv11-EMC model performs better in terms of accuracy compared to the YOLOv11 model in the concrete defect detection task; nevertheless, the model still suffers from the leakage problem, which may be due to the following reasons: firstly, the model has low sensitivity to small targets and defective regions with blurred edges. Concrete defects often present with small scales and irregular shapes, and the edge regions may be blurred due to the shooting angle or the influence of lighting, resulting in the insufficient feature extraction capability of the model and leakage detection; secondly, the sample distribution of the dataset may not be balanced enough. Despite the attempt to equalize the number of categories through data enhancement, the sample size of certain rare categories (e.g., whitening) is relatively small, and the model may not be able to learn the features of these categories sufficiently, leading to missed detection; thirdly, the data collected by UAVs have a large brightness range and varied scenarios. In certain extreme environments (e.g., bright light, shadows, rainy and foggy weather), the image quality may be affected, and the robustness of the model fails to fully adapt to these scenes. Therefore, these influences pose challenges for efficient concrete defect detection.

It is worth noting that although the accuracy metrics (P, R, mAP) of YOLOv11-EMC are all improved to different degrees compared to the original YOLOv11, the network depth and the number of parameters are also increased, which means that the deployment on some memory-constrained devices may be limited. Although our proposed enhanced deformable convolution module effectively improves the feature extraction ability for various shaped defects, it adds an offset learning branch compared to the normal convolution, which requires additional convolution operations to predict the offset coordinates of each convolution kernel sampling point. In addition, the sampling points of deformable convolution usually fall in non-integer positions, and the values of the sampling points need to be obtained by bilinear interpolation, and this interpolation operation introduces additional computational effort, which is more significant especially on high-resolution inputs or larger feature maps. We introduce the DS module to address the aforementioned issues. It can decrease computation and the number of parameters, but the model may extract too many redundant features in complex scenes and is unable to efficiently filter irrelevant information during feature fusion, which leads to an increase in parameters.

In terms of performance on the field test dataset, the improvement of the detection accuracy of our model in whitening defects is not obvious, not only because of the imbalance of the dataset, but also possibly because the features of whitening defects are usually manifested as weak differences in color rather than obvious texture or shape features, which reflects that there is still room for improvement in the sensitivity to color in YOLOv11-EMC.

## 6. Conclusions

In this study, an improved model based on enhanced dynamic convolution and deformable convolution is proposed, aiming to enhance the accuracy and adaptability of concrete defect detection. The results show that our model outperforms many other models in the YOLO family in terms of comprehensive performance on both public and UAV datasets. This effectively lowers misclassifications and under-detections while simultaneously increasing detection accuracy, confirming the high generalizability and robustness of our model. However, the model still has room for improvement in the identification of rust and efflorescence defects. In future research, we will focus on extending the dataset to cover different weather conditions and building types, further validating the model’s adaptability, and exploring strategies to enhance detection performance in complex environments to advance the practical applications of smart building maintenance and infrastructure management.

## Figures and Tables

**Table 1:** Experimental environment parameter information.

Environmental Parameter	Value
Operating system	Windows11
CPU	AMD Ryzen 5 5600
GPU	RTX4060 (10 GB)
Programming language	Python3.11
Deep learning framework	Pytorch2.01
RAM	16 GB

**Table 2:** Experimental environment parameter information.

Hyperparameters	Epoch	Batch	Lr0/f	Imgsz	Mosaic
Candidate value	100/300/500	8/16/32	0.01 ± 0.005	640/800/1000	1.0 ± 0.2

**Table 3:** Information on the optimal parameters of the experimental environment.

Hyperparameters	Epoch	Batch	Lr0/f	Imgsz	Mosaic
Candidate value	300	16	0.01	640	1.0

**Table 4:** Comparison of the main performance indicators of different models.

Model	P	R	mAP50	Params	GFLOPS	Layers
ResNet50-Faster R-CNN	0.817	0.601	0.668	25656988	35.1	58
VGG16-SSD	0.807	0.562	0.659	17823594	32.5	24
YOLOv5n	0.703	0.55	0.617	2509634	7.2	262
YOLOv8n	0.757	0.58	0.628	2691378	6.9	249
YOLOv9t	0.745	0.551	0.607	2661236	11.0	1219
YOLOv10n	0.749	0.576	0.624	2696756	8.2	368
YOLOv11n	0.712	0.594	0.632	2583322	6.3	238
Ours	0.81	0.596	0.666	3217730	6.3	386

**Table 5:** Comparison of the mAP values of different models for different kinds of defects.

Defect	YOLOv5n	YOLOv8n	YOLOv9t	YOLOv10n	YOLOv11n	Ours
Exposed Reinforcement	0.903	0.911	0.901	0.89	0.911	0.911
Rust Stain	0.453	0.412	0.375	0.419	0.425	0.467
Crack	0.574	0.577	0.579	0.58	0.581	0.607
Spalling	0.772	0.732	0.69	0.755	0.748	0.787
Efflorescence	0.35	0.309	0.326	0.31	0.343	0.354
Delamination	0.85	0.826	0.773	0.788	0.782	0.872

**Table 6:** Comparison of the inference verification performance of different models.

Model	P	R	mAP50	F1-Score	Params	GFLOPS	FPS
YOLOv11n	0.724	0.612	0.657	0.66	2583322	6.3	259
Ours	0.807	0.633	0.7	0.69	3217730	6.3	223

## Data Availability

The raw data supporting the conclusions of this article will be made available by the authors on request.
